# The Importance of Conditioned Stimuli in Cigarette and E-Cigarette Craving Reduction by E-Cigarettes

**DOI:** 10.3390/ijerph14020193

**Published:** 2017-02-15

**Authors:** Martijn Van Heel, Dinska Van Gucht, Koen Vanbrabant, Frank Baeyens

**Affiliations:** 1 Faculty of Psychology and Educational Sciences, KU Leuven, 3000 Leuven, Belgium; dinska.vangucht@thomasmore.be (D.V.G.); frank.baeyens@kuleuven.be (F.B.); 2 Department of Psychology, Thomas More University College, 3414 Antwerp, Belgium; 3 Interuniversity Institute for Biostatistics and Statistical Bioinformatics, KU Leuven and University of Hasselt, 3000 Leuven, Belgium; koen.vanbrabant@kuleuven.be

**Keywords:** e-cig, experimental study, conditioned stimuli, sensorimotor, aroma, visual cues, nicotine

## Abstract

This study examined the impact of four variables pertaining to the use of e-cigarettes (e-cigs) on cravings for tobacco cigarettes and for e-cigs after an overnight abstinence period. The four variables were the nicotine level, the sensorimotor component, the visual aspect, and the aroma of the e-cig. In an experimental study, 81 participants without prior vaping experience first got acquainted with using e-cigs in a one-week tryout period, after which they participated in a lab session assessing the effect of five minutes of vaping following an abstinence period of 12 h. A mixed-effects model clearly showed the importance of nicotine in craving reduction. However, also non-nicotine factors, in particular the sensorimotor component, were shown to contribute to craving reduction. Handling cues interacted with the level (presence/absence) of nicotine: it was only when the standard hand-to-mouth action cues were omitted that the craving reducing effects of nicotine were observed. Effects of aroma or visual cues were not observed, or weak and difficult to interpret, respectively.

## 1. Introduction

Many smokers say that they have the intention to quit and many make actual attempts, but the vast majority of these attempts is unsuccessful. Typically, no more than 3%–5% of smokers trying to quit without any assistance are still abstinent 6–12 months later [1]. Those smokers who do seek evidence-based assistance in their quit attempt are only two to three times more likely to become and remain abstinent in the long-term [2]. Assistance includes medically approved smoking cessation aids and professional quit-smoking assistance, such as quit-smoking medication, short-term pharmaceutical Nicotine-Replacement Therapy (NRT) and/or behavioral counseling.

Tobacco Harm Reduction (THR) may prove a viable alternative for those smokers who are not able or not willing to cease all tobacco and/or nicotine consumption. THR refers to any actions taken to reduce the substantial health risks associated with tobacco smoking, such as educating people about the risks of different sources of nicotine, and providing smokers with the opportunity to switch from tobacco smoking to the use of low-risk nicotine products [3,4,5,6] including smokeless tobacco (e.g., Swedish snus), long-term pharmaceutical NRT, or more recently, e-cigs. A unique characteristic of e-cigs is that, while acknowledging the importance of efficient nicotine delivery, they also aim to address the behavioral and sensory aspects of smoking. At least at face value, e-cigs appear to provide good mimicry of many of the crucial Conditional Stimuli (CSs) related to the act of smoking tobacco cigarettes (for example, hand-to-mouth action, visual cues, sensorimotor cues of inhaling and exhaling, throat hit, tobacco flavor) [3,6].

Taking into account that one pack-year of smoking equals over 70,000 puffs, a typical 40 year-old smoker will have experienced 1–2 million trials in which nicotine administration and its rewarding effects has been contingently paired with a well-specified set of proximal CSs (hand-to-mouth action, visual cues, sensorimotor cues of inhaling/exhaling, throat hit, flavor/smell) and a less well-specified set of more distal CSs (location, time-of-the-day, preceding or concurrent activities, social context, internal/emotional cues). Exposure to these cues (when smoking a new tobacco cigarette) or to perceptually highly similar cues (for example, when using an e-cig) can thus be expected to tap into and activate an already well-established associative memory network consisting of smoking/nicotine-associated cues linked to previously experienced nicotine effects. The critical involvement of such non-pharmacological CSs associated with smoking in the reinforcing and craving reduction effects of nicotine has been documented in both animal and human research [7,8,9,10,11,12,13].

Animal research suggests that the primary reinforcing effects of nicotine are relatively weak compared to other substances, such as cocaine or heroin [7,8,9]. There is strong evidence that animal nicotine self-administration not only depends on the primary rewarding effects of response-contingent nicotine delivery but also on the conditioned rewarding effects of non-nicotine cues (e.g., visual or auditory stimuli) that are associated with nicotine delivery [14]. Moreover, nicotine has also been shown to act as a (non-contingent) reinforcement enhancer of other unconditioned or conditioned (nicotine-associated) reinforcers, synergizing in a multiplicative rather than an additive fashion with nicotine in the acquisition and maintenance of nicotine self-administration [15].

In the same vein, human data on nicotine craving reduction and smoking satisfaction point to the critical role of non-pharmacological CSs [10,11,12,13]. For example, Rose and colleagues [12] designed an experimental procedure allowing assessment of the relative contribution of the smoking ritual and of nicotine administration on cigarette craving reduction after a 12-h overnight abstinence period. Smokers received intravenous injections of either nicotine or saline (control), and, orthogonal to this manipulation, they were or were not allowed to simultaneously smoke a denicotinized cigarette. The resulting four experimental conditions were compared to a condition in which participants smoked their usual brand of nicotine-containing cigarettes. Cigarette craving was assessed prior to and 30 and 60 min after the experimental manipulation. The relieving effects solely due to the intravenous nicotine administrations were observed to be substantially lower than the satiation resulting from smoking a usual brand cigarette. The sensorimotor stimuli alone (provided by smoking the denicotinized cigarette) were observed to result in a similarly small but reliable relief of the craving to smoke. Only when intravenous nicotine and sensorimotor components were combined (nicotine injections plus smoking denicotinized cigarette), a relief level similar to the craving reduction resulting from smoking a usual brand cigarette was achieved.

In this condition of the study by Rose and colleagues [12], the smokers were exposed to most of the stimuli associated with regular smoking—participants smoked an actual cigarette, be it denicotinized—while simultaneously obtaining nicotine, which probably created an optimal situation for craving reduction. As argued above, vaping an e-cig creates a sensorimotor experience that is substantially similar (but not identical) to that resulting from smoking: it exposes the user to many (but not all) of the critical stimuli associated with the ritual of smoking while intermittently providing her with doses of nicotine. Vaping essentially emulates the condition of the study by Rose and colleagues [12] in which participants were allowed to smoke while they received doses of nicotine intravenously. Some studies have found that the nicotine delivery resulting from vaping may be less efficient (less and more gradual delivery of nicotine to the blood and brain) than nicotine delivery resulting from tobacco smoking [16,17]. However, in other studies using more advanced (high-wattage) devices, higher nicotine concentrations in the e-liquid and experienced users, or a combination of those, nicotine delivery profiles approaching or even surpassing those of combustible tobacco cigarettes have been observed [18,19,20]. Despite this heterogeneity in nicotine delivery profiles, and despite the fact that the non-nicotine cues provided by vaping are similar but not identical to those of smoking combustibles, multiple experimental studies have convincingly and unanimously shown that vaping can indeed lead to craving reduction.

Vansickel [21] allowed participants to take 10 puffs of an e-cig with 16 or 18 mg/mL nicotine, to smoke (10 puffs) a combustible tobacco cigarette, or to sham smoke (holding an unlit tobacco cigarette) after a 12-h abstinence period. The reduction in desire to smoke was reliable for the e-cigs with nicotine but weaker than for tobacco cigarette smoking, and absent after sham smoking. Bullen and colleagues [16] found that the desire to smoke after overnight abstinence was significantly reduced after five minutes of vaping an e-cig with 16 mg/mL nicotine, but also with an e-cig containing 0 mg/mL nicotine. However, the reduction found in the 16 mg/mL nicotine condition was significantly stronger than in the no-nicotine condition. The same conclusions were reached by Dawkins and colleagues [22] who presented their participants with either an e-cig containing 0 mg/mL or 18 mg/mL nicotine to vape for five minutes. An important difference is that the abstinence period here was limited to one hour, which indicates the robustness of the reduction findings over different durations of smoking abstinence. Dawkins and Corcoran [23] recruited experienced vapers and asked them to abstain overnight from all tobacco/nicotine products. In the morning, they were presented with a first generation e-cig containing 18 mg/mL nicotine, from which they were allowed to take 10 puffs. As a result, the nicotine craving and urge to smoke were significantly reduced in these experienced vapers. In a group of smokers without any previous experience with vaping and using a more advanced open-system e-cig with e-liquid containing 18 mg/mL of nicotine, Adriaens and colleagues found that five minutes of vaping resulted in immediate craving reduction after four hours of abstinence that was equally strong as the craving reduction obtained by smoking a tobacco cigarette [24]. In summary, earlier studies clearly established the craving reduction effects of vaping in controlled laboratory trials; these effects were sometimes also present when using e-cigs containing no nicotine, be it to a lesser degree. This suggests that nicotine cannot be solely accountable for the observed craving reduction. The question then becomes which particular aspects of the smoking ritual (CSs) are responsible for this craving reduction.

Dawkins and colleagues [25] presented participants with either a red or white cig-a-like nicotine-containing (18 mg/mL) “tobacco” flavored e-cig after a 10-h abstinence period. Their results suggest that the visual appearance of an e-cig has an effect on cigarette craving reduction. They found that the more the e-cig resembled a regular cigarette (white color), the stronger the craving reduction. However, this effect was only found in people without prior experience with e-cigs [25]. As discussed above, the study of Rose and colleagues [12] suggested that handling a (tobacco) cigarette, including the act of inhaling and exhaling, also contributes to craving reduction; it remains to be seen, however, whether or not this finding can be replicated in the context of vaping. Finally, there is some indirect experimental evidence suggestive of the involvement of aroma as important non-nicotine cues: Audrain-McGovern and colleagues [26] demonstrated that flavored e-cigs (“green apple” or “chocolate” vs. unflavored) resulted in a subjectively more rewarding vaping experience, whereas Goldenson and colleagues [27] showed that nicotine-free and nicotine-containing (6 mg/mL) e-cigs produced greater appeal when containing sweet flavors (e.g., peach, blackberry) than when containing non-sweet flavors (e.g., tobacco, menthol) or no flavor, but the authors of both experimental studies did not assess whether or not this had any impact on the craving reduction potential of vaping. Altogether, these lab studies suggest that sensorimotor stimuli, as well as visual and also flavor cues, may contribute to the effectiveness of the e-cig in terms of craving reduction [12,25,27].

The aim of this experimental study was to examine the impact of four variables pertaining to the use of e-cigs on craving for tobacco cigarettes and for e-cigs after an overnight abstinence period. The four variables were the nicotine level (0% vs. 3.6% nicotine), the sensorimotor component (e-cig handled by participants vs. held by a unipod), the visual aspect (no visual restrictions vs. blindfold), and the aroma (tobacco flavor vs. apple flavor) of the e-cig. These variables were manipulated in an orthogonal manner, resulting in a between-group 2 × 2 × 2 × 2 factorial design (16 experimental conditions). The study consisted of two lab sessions (session 1: intake plus baseline measurements; session 2: experimental vaping session) that were separated by a (approximately) one-week period during which participants, who were all naïve about e-cigs, could familiarize themselves with vaping.

The effects of nicotine and of the non-nicotine factors were investigated as exploratory. No specific hypotheses were put forward concerning the (relative) strength of possible main effects or with respect to the existence or importance of interactions between the manipulated variables. In general, however, we expected (a) an overall craving reduction following use of the e-cigs; (b) which would show up in the nicotine (3.6% nicotine) as well as in the no nicotine (0% nicotine) conditions but (c) which was hypothesized to be weaker in the no nicotine conditions than in the nicotine conditions. Furthermore, we predicted (d) a facilitating influence from the presence of handling cues (manual handling better than unipod handling), visual cues (unrestricted vision better than blindfolded), and aroma (tobacco flavor better than apple flavor).

## 2. Materials and Methods

### 2.1. Participants

All subjects gave their informed consent for inclusion before they participated in the study. The protocol was approved by the medical ethical committee of KU Leuven (ML10158 B322201420221). Participants were mainly recruited through flyers and online advertisements from the local community in Leuven (Belgium) and its surroundings, between February 2014 and February 2015. The following selection criteria were used to recruit the participants: smoking about 10 factory-made cigarettes per day during the past three years and not having any experience with vaping. Additionally, participants were required not to be currently attempting to quit smoking, nor to have the intention to quit smoking in the near future. No smoking cessation methods were used by any of the participants. However, all participants had to be interested in trying out a healthier alternative to traditional tobacco cigarettes. People with diabetes or severe allergies were excluded from this experiment as well as people with asthma or other airway disorders. Furthermore, people that suffered from psychiatric problems, chemical dependence other than nicotine, high blood pressure or other cardiovascular diseases were excluded from the study. Finally, only non-pregnant and non-breastfeeding women could participate. Participants decided through self-selection (and self-report) if they were eligible.

### 2.2. Materials and Equipment

#### 2.2.1. CO Measurement

The amount of carbon monoxide (CO) in the exhaled breath was measured using a “piCO + Smokerlyzer” (Bedfont Scientific, Maidstone, UK). All measurements were carried out in accordance with the published instructions [28]. The apparatus used in this study was calibrated on 13 March 2014, a few weeks prior to the onset of our trials. The measurement of CO during the information session served as a baseline for future measures of CO.

#### 2.2.2. Electronic Cigarettes

The e-cigs used for this study were disposable “Fling” models manufactured by the company White Cloud (Tarpon Springs, FL, USA). This type was selected after comparing several models and based on reviews of disposable e-cigs on several consumer fora. The chosen e-cigs consisted of a non-rechargeable battery, an atomizer and a liquid container serving as the mouthpiece. The tip of the e-cig contained a red LED that lights up when the vaper inhales. According to the product documentation provided by White Cloud, these e-cigs contained 0%, 2.4% or 3.6% nicotine, 84.6%–92% propylene glycol (PG) and glycerol (VG) (80/20 PG/VG ratio) and 8.0%–10.0% flavors. The experimental e-cigs contained either no-nicotine (0%) or nicotine (3.6%). Half of the experimental e-cigs were apple flavored (“Bad Apple”), and the other half were tobacco flavored (“Tobacco Regular”). The try-out e-cigs contained nicotine (2.4%) and were tobacco flavored (“Tobacco Regular”).

#### 2.2.3. Unipod and Blindfold

**Figure 1:**
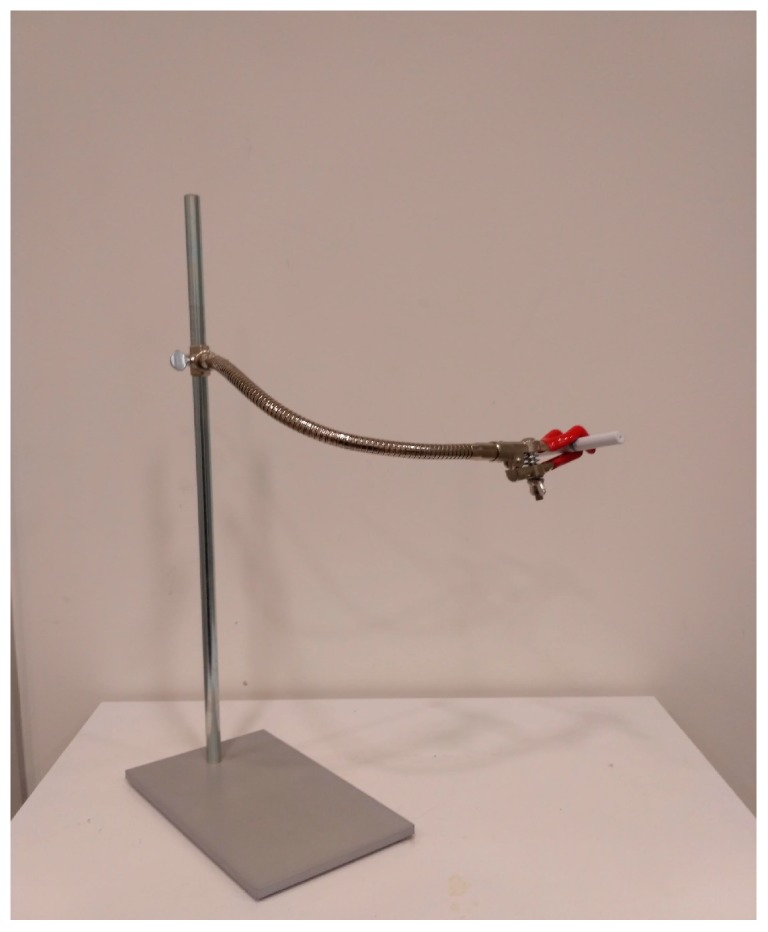
The unipod used in the Unipod condition.

In the condition where participants could not handle the e-cig themselves, a unipod with a flexible arm with a three-finger clamp was used to hold the e-cig (see Figure 1). Before the five-minute vaping trial, the participants were given the opportunity to put the arm with the e-cig in a position that they were comfortable with. In the blind condition, participants were blindfolded so that they could not see anything. The lights were also turned off in order to minimize visual stimulation.

#### 2.2.4. Questionnaires

Three questionnaires were used to obtain general information on participants’ characteristics; these focused on the smoking history, the demographics, and the nicotine dependence, respectively. This last questionnaire was the “Fagerstrom Test for Cigarette Dependence” (FTCD) [29].

Visual Analogue Scales (VASs) were used to measure craving for an e-cig and for a tobacco cigarette. Each scale was 10 centimeters long (scores ranged from 0 to 100). The exact questions were (translated from Dutch): “How strongly do you feel like having an e-cig at this moment?” and “How strongly do you feel like having a regular cigarette at this moment”? The left side of the scale was accompanied by the label “not feeling like having (a cigarette/e-cig) at all”, and the right side of the label “very strongly feeling like having (a cigarette/e-cig)”. Two Tobacco Craving Questionnaires (TCQ) [30] were used, namely one for the tobacco cigarette [31] and one for the e-cig [25]. The scores on the TCQ range from 12 to 60. Across participants, the order in which these TCQs and VASs were presented was counterbalanced: in both the nicotine and no nicotine condition, half of the participants started with the questions about cigarettes, while the other half started with the questions about e-cigs. The craving for cigarettes and e-cigs were assessed separately because it seems plausible that craving for cigarettes and for e-cigs can differ from each other. In addition, a previous study by Adriaens and colleagues showed that when smokers switch to vaping, craving can actually shift from cigarettes to e-cigs [24]. 

In addition, participants were also asked to fill out three other questionnaires. The first one assessed how participants subjectively perceived each of the experimental manipulations and obtained information on the subjective importance of the manipulated variables (the flavor of the e-cig, the throat hit, the method of handling the e-cig, and the visual aspects). More specifically, the questions assessed whether the participants liked or disliked the way in which the variable was manipulated in his/her specific condition. Furthermore, the personal relevance of the variable was assessed. The second questionnaire specifically aimed at the comparison of the e-cig with the tobacco cigarette, relating to participants’ past week experience with the three try-out e-cigs. The questions dealt with the looks, the handling, the smoke/vapor, the taste, the throat hit, the overall experience and the health risks. All questions were asked with respect to e-cigs and also with respect to tobacco cigarettes. The third and last questionnaire focused on participants’ last week use of the e-cig. This questionnaire addressed questions such as their intention to quit smoking and what effect the use of the e-cig had on their smoking behavior. Additionally, participants were also asked about their overall experience with the e-cig and the advantages and disadvantages of both the regular brand tobacco cigarette and the e-cig. Finally, they were asked about how they thought their e-cig use would evolve in the future. Two weeks after the experimental session, they received an e-mail with a follow-up questionnaire on their vaping and smoking behavior. The data from these additional questionnaires was not used and will not be reported in the context of the present report.

### 2.3. Procedure

All meetings were scheduled using a doodle link from Google (Mountain View, CA, USA). If participants were students, the participants’ recruitment system of the Faculty of Psychology and Educational Sciences of KU Leuven was used. The two sessions were planned with a time interval ranging from four days up to two weeks, with an average length of eight days.

#### 2.3.1. First Session

Immediately upon arrival in the lab, the baseline amount of CO in the exhaled breath of the participants was measured. After this, they attended an information session on vaping, the components of the e-cig, how to use the e-cig, and legislation. Afterwards, the informed consent was introduced and participants were informed that they could terminate their involvement at any point in time during the whole study. After agreeing to these terms, participants also completed the questionnaire that includes the smoking history, the demographics, and the nicotine dependence. At the end of this session, three try-out e-cigs were given to the attendees (in exchange for a 20 euro warranty) so that they could familiarize themselves with vaping for about one week, the exact duration depending on when the second appointment was scheduled. Participants were not asked to quit smoking tobacco cigarettes, so, if they wanted, they could use both. Participants took home a summary containing information about e-cigs, components and the working of the e-cig, the reduction of risks compared with regular cigarettes, a list of references to scientific publications documenting these reduced risks, and names of forums where they could read experiences of e-cig users. Before participants left, the experimenter reminded them of the date of their follow-up session, stressing the instruction to abstain from smoking and vaping at least 12 h before that next session.

#### 2.3.2. Second Session

**Figure 2:**
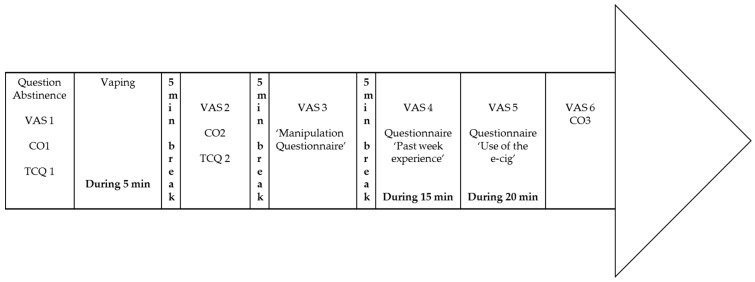
Timeline of the second (experimental) session. Please provide clearer figure.

Approximately one week later, the participants returned to the same location. As indicated in Figure 2, the participants were asked upon arrival how long they had abstained from smoking/vaping, and they were presented with the two craving VASs (1). Next, the amount of exhaled CO (1) was measured, after which they were asked to fill out the two TCQs (1). Upon completion of these questionnaires, participants were allowed to vape the experimental e-cig for five minutes. They did not receive any instructions regarding the number or duration of puffs from the e-cig. The experimental manipulation determined the kind of e-cig they could vape (no-nicotine or 3.6% nicotine; tobacco flavored or apple flavored e-cigs), the method of handling (handle the e-cig themselves or e-cig held by a unipod), and whether visual stimuli were present or not (unrestricted vision or blindfolded). Information about flavor and nicotine content was printed on the e-cig but had been made illegible by crossing the printed text out with a felt pen.

Immediately after these five minutes of vaping, the VASs (2), TCQs (2) and CO (2) measurement were presented again. When participants had completed all questionnaires, a five-minute break was scheduled. Next, they were asked to fill out the VASs (3) for the third time. At this moment, participants also filled in the instrument that assessed the subjective perceptions of the manipulated variables. After another five-minute break, participants were given 15 min to fill out the fourth set of VASs (4) followed by the questionnaire focusing on the comparison between the characteristics of smoking tobacco cigarettes and using an e-cig. In the subsequent period (20 min), participants first indicated their craving for either an e-cig or a regular tobacco cigarette, using the fifth set of VASs (5). Additionally, they completed the questionnaire about their use of the e-cig during the try-out week. This did not take the full 20 min, which left the opportunity to provide information about the online follow-up questionnaire and to return their 20 euro warranty. Finally, participants were asked to fill out a sixth and last set of VASs (6) and provided a final CO (3) measurement. They were allowed to take the remaining e-cig home. 

## 3. Results

### 3.1. Statistical Analyses

Mixed-effects models were used to evaluate the craving reduction measured via the four different instruments (i.e., TCQ, e-cig TCQ, VAS, and e-cig VAS). These mixed-effects models allowed a random intercept for all subjects to control for the dependencies that arise in the dataset due to the multiple measurements per subject. The Measurement Moments were always included as a factor in the mixed-effects models. This factor only had two levels for all the TCQ measures and six levels when applied to the VAS scales. Throughout the analyses, we always followed the same structure: first, test for the effect of the Measurement Moments and afterwards include the effect of the manipulated Nicotine Level. Afterwards, we tested for the effect of the three additionally manipulated variables (i.e., Handling, Visual Cues, and Aroma) in separate three-way interaction models. Higher-order interactions were not considered due to a lack of theoretical expectations and a lack of power to detect these eventual higher order interactions. Likelihood-ratio-tests (LRT) were used for inferences on multiple parameters and Wald tests for inferences on single parameter tests. All analyses were performed in R version 3.3.1 (R Foundation for Statistical Computing, Vienna, Austria) [32], the mixed-effects models were performed in the lme4 version [33], *p*-values were calculated with the package lmertest version [34], and plyer [35] and reshape2 [36] packages were used for general data management.

### 3.2. General Characteristics

Participants (*n* = 81; 45.7% females) were between 18 and 68 years old (M = 29.8; SD = 13.2), and smoked on average 13.4 tobacco cigarettes a day (SD = 5.61). They started smoking between 12 and 21 years of age (M = 16.10; SD = 1.89). Sixty-six participants (56.8%) wanted to smoke less. The number of past quit attempts ranged from zero up to 10 attempts. The scores on the FTCD [29] showed that 29.1% fell in the “very low dependent” category (score 0–2), 27.8% fell in the “low dependent” category (score 3–4), 21.5% were situated in the “moderate dependent” category (score 5), 20.3% were situated in the “high dependent” category (score 6–7) and 1.3% fell in the “very high dependent” category (score 8–10). On average, the participants had a score of 3.89 (SD = 2.08), which indicates an average “low dependency”. The average dependency did not significantly differ between experimental groups (Nicotine: M = 4.00, No-Nicotine: M = 3.78, *t* (77) = −0.479, *p* = 0.633; Visual: M = 3.67, Blind: M = 4.10, *t* (77) = −0.927, *p* = 0.357; Manual: M = 3.90, Unipod: M = 3.88, *t* (77) = 0.048, *p* = 0.962; Tobacco Aroma: M = 3.95, Apple Aroma: M = 3.82, *t* (77) = 0.288, *p* = 0.774).

### 3.3. CO Measures

**Figure 3:**
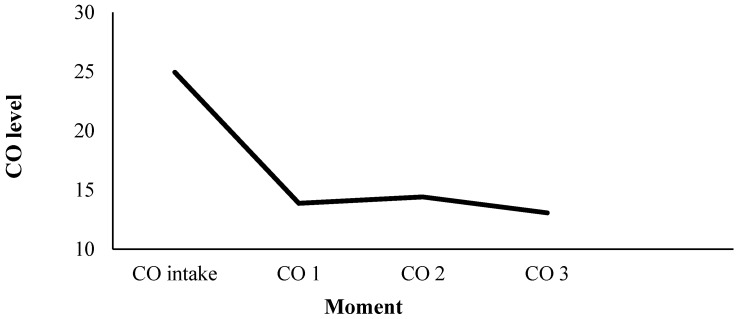
Carbon monoxide (CO) measurements over time.

The average self-reported duration of abstinence was nine hours and 12 min. The CO measures were included as an objective indicator of the length of the abstinence period. Across all conditions, a significant reduction in CO levels was observed between the measurement at the intake session and the measurement at the start of the test session using contrasts (Intake CO: M = 24.69, SD = 1.35; CO1: M = 13.74, SD = 1.06; *t* (243) = −10.31, *p* < 0.001). During the 70 min of the test session, the CO levels did not significantly decrease any further, nor did they reliably increase (CO1 − CO3 = 0.79, SD = 1.06; *z* = 0.744, *p* = 0.457) (see Figure 3).

### 3.4. Tobacco Craving Questionnaire

As discussed in the procedure, we used the TCQ to measure tobacco cigarette craving as well as the craving for an e-cig. We will start with the discussion of the results for the tobacco cigarette craving, and afterwards will proceed with the e-cig craving. Because the TCQ was only measured on two occasions, the discussed craving reduction will always refer to the difference between the pre and post vaping measurements.

#### 3.4.1. Cigarette Craving (TCQ)

**Figure 4:**
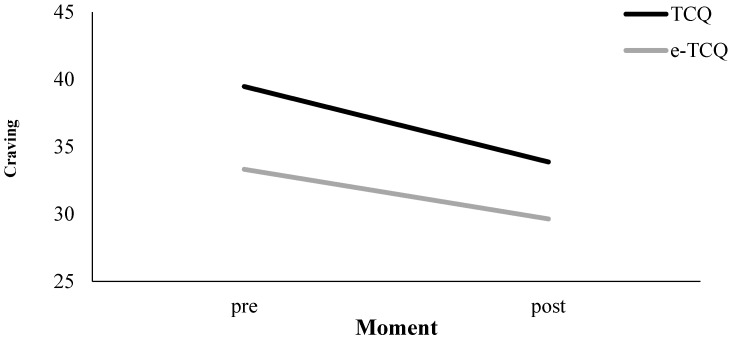
TCQ craving measurements pre and post vaping: tobacco cigarette craving (TCQ) and e-cig craving (e-TCQ).

**Figure 5:**
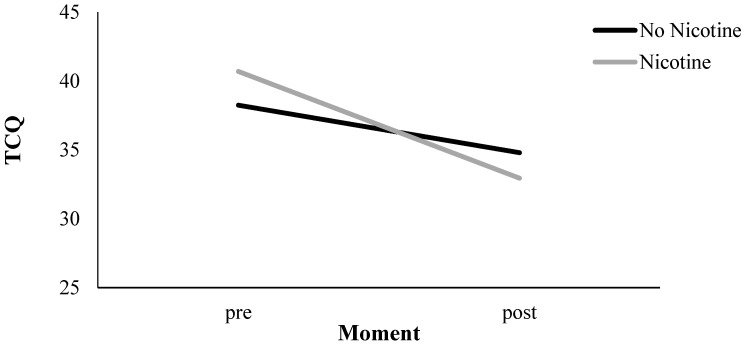
Tobacco cigarette craving (TCQ) pre and post vaping: effect of Nicotine Level.

Results are presented in Table 1 and in Figure 4. Across Nicotine Level conditions, there was an overall effect of Measurement Moment (*p* < 0.01), implying a craving reduction for tobacco cigarettes of 5.51 points on the TCQ scale (*p* < 0.01). In addition, there was an interaction between Nicotine Level and Measurement Moment for tobacco cigarette craving (*p* < 0.01). In the nicotine group, we observed a reduction of 7.75 TCQ points (*p* < 0.01), and, in the no-nicotine group, we observed a decline of 3.34 TCQ points (*p* < 0.01; see Figure 5). To investigate the influence of the three other variables (i.e., Handling, Aroma, and Visual Cues) on tobacco cigarette craving measured by the TCQ, we included these variables in separate three-way interaction models in addition to the Measurement Moment and Nicotine Level. None of these three variables, however, contributed significantly to the observed craving reduction (i.e., Handling, *p* = 0.18; Aroma, *p* = 0.41; Visual Cues, *p* = 0.38). In the next paragraph, we describe the results of the same analyses on the TCQ that measured e-cig craving.

#### 3.4.2. E-Cig Craving (e-TCQ)

**Figure 6:**
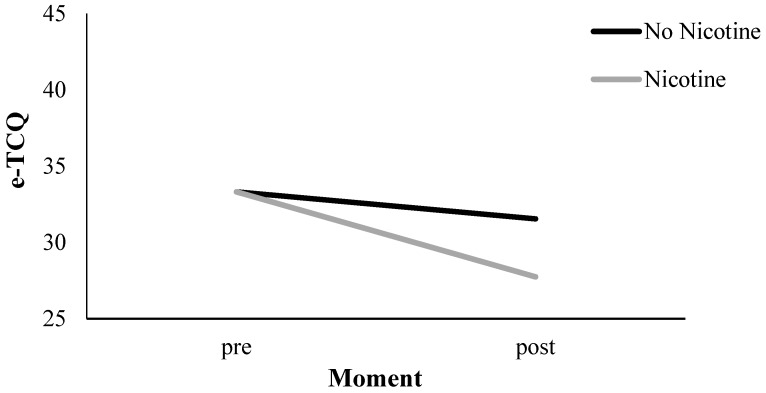
E-cig craving (e-TCQ) pre and post vaping: effect of nicotine level.

Results are presented in Table 1 and in Figure 4. Across Nicotine Level conditions, there was an overall effect of Measurement Moment (*p* < 0.01), implying a craving reduction for e-cigs of 3.60 points on the TCQ scale (*p* < 0.01; see Figure 4). In addition, there was an interaction between Nicotine Level and Measurement Moment for e-cigarette craving (*p* < 0.05). In the nicotine group, we observed a reduction of 5.57 TCQ points (*p* < 0.01), and, in the no-nicotine group, we observed a decline of 1.68 TCQ points that was non-significant (*p* = 0.14; see Figure 6). To investigate the influence of the three other variables (i.e., Handling, Aroma, and Visual Cues) on e-cig craving measured by the TCQ, we included these variables in separate three-way interaction models in addition to the Measurement Moment and Nicotine Level. None of these three variables, however, contributed significantly to the observed craving reduction (i.e., Handling, *p* = 0.85; Aroma, *p* = 0.78; Visual Cues, *p* = 0.11). 

### 3.5. Visual Analogue Scales

The VAS measures were obtained at six points in time, including one pre and five post vaping measurements. Hence, the analyses of the tobacco cigarette and e-cig VASs can provide insight into the course of craving beyond the pre and post vaping points. First, a correlation with the TCQ was calculated to assess the concurrent validity of the craving measures. For tobacco cigarette craving measures, the Pearson correlation pre-vaping was 0.62 (*p* < 0.01) and 0.79 (*p* < 0.01) post-vaping. The same was done for the e-cig craving measures: the correlation was 0.53 (*p* < 0.01) pre-vaping and 0.65 (*p* < 0.01) post-vaping. These correlations imply a moderate to high degree of concurrent validity. In the next section, we will discuss the results of the tobacco cigarette craving VAS and the e-cig craving VAS separately. We start with the results of the tobacco cigarette craving data.

#### 3.5.1. Cigarette Craving (VAS)

Results are presented in Table 2. Across Nicotine Level conditions, there was an overall effect of Measurement Moment on tobacco cigarette craving reduction (*p* < 0.01). Two additional tests were of interest with respect to this overall effect, a first assessing craving reduction from the pre to the first post vaping Measurement Moment, a second assessing craving reduction from the pre vaping moment to the five post vaping Measurement Moments combined. With respect to the first question, we observed a decline in the craving of 19.43 points between the pre and first post vaping Measurement Moment (*p* < 0.01). With respect to the second question, we observed a reduction of 14.88 points on the craving scale (*p* < 0.01).

There was little evidence of an interaction effect between Nicotine Level and Measurement Moment on the craving reduction for tobacco cigarettes (*p* = 0.09). However, when comparing the pre and post vaping measurements, the nicotine group showed a decline of 26.76 points (*p* < 0.01), whereas the no-nicotine group showed a decline of 12.10 points (*p* < 0.01). The difference in decline between both Nicotine Level groups from pre to post measurement was significant, *t* (1) = −3.18, *p* < 0.01. A contrast analysis in the nicotine group between the pre vaping measurement moment and a compound of all five post vaping measurements indicated a reduction of 18.79 points on the VAS scale (*p* < 0.01). In the no-nicotine group, we observed a reduction of 10.97 points for the same contrasts (*p* < 0.01).

**Figure 7:**
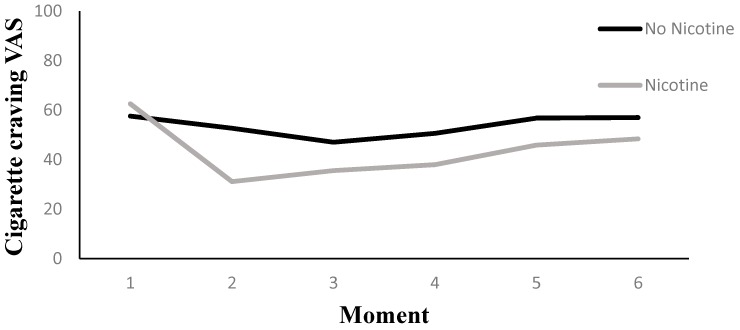
The interaction between moment and nicotine level in the Unipod condition for cigarette craving.

We tested the effect of the three additionally manipulated variables (i.e., Handling, Aroma, and Visual Cues) in three separate models, which included a three-way interaction between Measurement Moment, Nicotine Level, and the additionally manipulated variable. The models that tested for the triple interaction, involving Aroma (*p* = 0.31) and Visual Cues (*p* = 0.23), remained non-significant. Only the model with the triple interaction involving Handling (*p* = 0.09) contained enough evidence to further investigate in detail. In order to break down this triple interaction, we performed some additional analyses showing that there were no differential effects of Nicotine Level on craving reduction when the subjects could manually handle their e-cig (*p* = 0.87), whereas there were differential effects of Nicotine Level on craving reduction when the subjects could not handle their e-cig manually (Unipod) (*p* ≤ 0.01). When the unipod was used, the nicotine group had a pre to post craving reduction that was 26.50 points stronger in comparison to the no nicotine group, *t* (1) = 4.36, *p* < 0.01. This difference stayed significant across the next four post vape Measurement Moments but declined in strength when the Measurement Moment was further away from the vaping experience (see Figure 7).

#### 3.5.2. E-Cig Craving (VAS)

Results are presented in Table 2. Across Nicotine Level conditions, there was an overall effect of Measurement Moment on e-cig craving reduction (*p* < 0.01). Two additional tests were of interest with respect to this overall effect, a first assessing e-cig craving reduction from the pre to the first post vaping measurement moment, a second assessing e-cig craving reduction from the pre vaping moment to all the five post vaping Measurement Moments combined. With respect to the first question, we observed a decline in e-cig craving of 11.14 points between the pre and post vaping Measurement Moment (*p* < 0.01). With respect to the second question, we observed a reduction of 7.07 points on the e-cig craving scale (*p* < 0.01).

As was the case for the tobacco cigarettes craving, there was little evidence for an interaction effect between Nicotine Level and Measurement Moment on e-cig craving reduction (*p* = 0.11). However, when comparing the pre and post vaping VAS measurements, the nicotine group showed a decline of 15.42 points (*p* < 0.01), and the no-nicotine group showed a decline of 6.86 points (*p* < 0.01). The difference in decline between both nicotine groups from pre to post measurement was not significant, however, at *t* (1) = 1.85, *p* = 0.07. A contrast analysis in the nicotine group between the pre vaping measurement moment and a compound of all five post vaping measurements combined indicated a reduction of 8.40 points on the e-cig craving scale (*p* < 0.01). In the no-nicotine group, we observed a reduction of 5.75 points for the same contrasts (*p <* 0.05).

**Figure 8:**
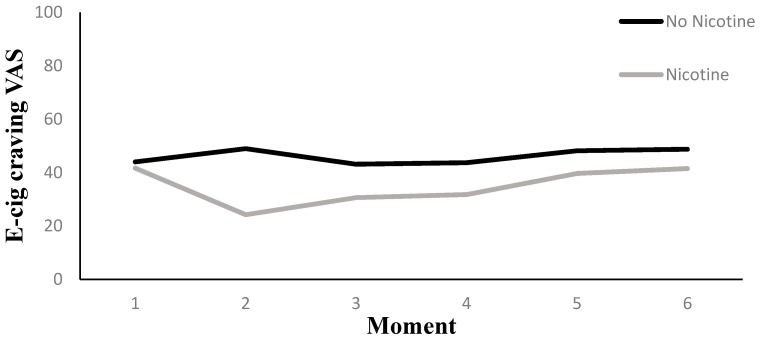
The interaction between moment and nicotine level in the visually unrestricted condition for e-cig craving.

We tested the effect of the three additionally manipulated variables (i.e., Handling, Aroma, and Visual Cues) in three separate models, which included a three-way interaction between Measurement Moment, Nicotine Level, and the additionally manipulated variable. The model that tested for the triple interaction involving Aroma was non-significant (*p* = 0.59). The triple interactions involving Visual Cues (*p* = 0.08) or the effect of Handling (*p* = 0.09) contained enough evidence, however, to further investigate in detail. The triple interaction involving Visual Cues implied that when the subjects were visually unrestricted, but not when they were blindfolded (*p* = 0.61), a difference in e-cig craving reduction between both nicotine levels was observed (*p* < 0.05). Namely, when participants were visually unrestricted, the nicotine group had a craving reduction from pre to post vape that was 22.43 points stronger than the no-nicotine group, *t* (1) = 3.44, *p* < 0.01, and no craving reduction was observed for the latter (see Figure 8).

**Figure 9:**
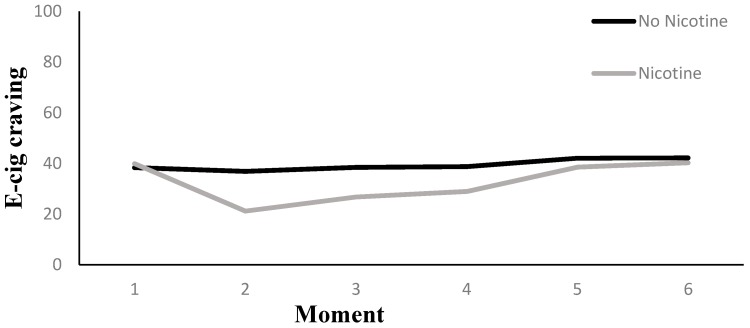
The interaction between moment and nicotine level in the Unipod condition for e-cig craving.

The triple interaction involving the Handling variable was due to the fact that there was an effect of Nicotine Level when the e-cig was offered via a unipod (*p* = 0.06), but not when it could be handled manually (*p* = 0.19). More specifically, when the unipod was used, the nicotine group had stronger e-cig craving reduction at the first and second post vape measurement than the no-nicotine group. The difference at the first follow-up was 17.18, *t* (1) = 2.72, *p* < 0.01, and 13.18, *t* (1) = 2.09, *p* < 0.05, at the second follow-up (see Figure 9). 

## 4. Discussion

In the present study, it was investigated which factors contributed to cigarette and e-cig craving reduction after a 12-h abstinence period. More specifically, the relative contribution of nicotine and non-nicotine (conditioned) factors were taken under scrutiny. Apart from the nicotine concentration of the e-cigs, we manipulated three non-pharmacological smoking/vaping-associated CSs for which there was some preliminary experimental evidence of their potential involvement in craving reduction [12,25,27]: Handling (Manual/Unipod), Visual Cues (visual/blindfold) and Aroma (apple/tobacco). The induction of craving, by means of an abstinence period prior to the test session, was successful. The CO level did significantly decline from the intake to the beginning of the test session. This indicates a period of abstinence from smoking in which (e-) cigarette craving is expected to build up.

The first experimental finding is that a decrease in craving was obtained regardless of whether nicotine was present or not. We observed reliable craving reduction when the e-cig contained no nicotine, but the decrease was significantly stronger when nicotine was present. With this finding, we replicated earlier findings by Bullen and colleagues and Dawkins and colleagues [16,22]. This superiority of nicotine-containing e-cigs showed up in both craving measures (TCQ and VAS) and both with respect to cigarette craving and e-cig craving. What this study does not speak to is the specific mode of action of nicotine with respect to craving reduction. The most obvious route is, of course, the direct pharmacological central nervous system effects of nicotine (nicotine-acetylcholine-glutamate- gamma-aminobutyric acid (GABA)-dopamine neural circuitry promoting nicotine reward, dependence, and withdrawal) [37]. Another mechanism may be the airway sensory impact of nicotine (“throat hit”), which has been shown to contribute to the conditioned rewarding effects (pleasantness, desirability) of (tobacco) cigarette puffs independently from the dopaminergic central nervous system effects [38], as well as to the potential of e-cigs to evoke a self-reported readiness to switch from tobacco smoking to e-cigs in smokers [39]. Our finding confirms the important role of nicotine in craving reduction, but, at the same time, it indicates that other, conditioned factors may play a role as well [14].

A second experimental finding resulted from the exploratory analysis of the VAS measures. The presence or absence of handling cues proved to interact with the presence or absence of nicotine with respect to the amount of craving reduction, both with respect to cigarette craving and e-cig craving. When the cues related to the standard hand-to-mouth action were present, the craving reduction was obtained irrespective of the presence or absence of nicotine. Only when a unipod was used so that the habitual hand-to-mouth sensorimotor stimuli were eliminated were the craving reducing effects of nicotine observed. A plausible explanation for this observation is that it is due to a floor effect with respect to the maximum observable craving reduction in this artificial experimental lab situation. Moreover, it should be interpreted with caution, as it only showed up in the VAS data, not in the TCQ. One possible explanation of the latter is the fact that the TCQ assesses more and different aspects of craving (e.g., the ability to limit one’s smoking, when a cigarette is available). For example, it may be possible that the hand-to-mouth cues influence the extent to which the participants feel like smoking, but do not influence all aspects of the craving construct as measured by the TCQ (namely, “emotionality”, “expectancy”, “compulsivity”, and “purposefulness”).

The third experimental finding resulting from the exploratory analyses was an effect of the visual cues on e-cig craving reduction. When participants were blindfolded, craving reduction was obtained irrespective of the presence or absence of nicotine. Only when participants had unrestricted vision did the craving reducing effects of nicotine come into play. This pattern of results is at the same time counterintuitive, hard to explain given previous research [26], and does not easily fit within the framework of classical conditioning and the role of conditioned stimuli. However, it must be stressed that this was only observed with respect to e-cigarette craving, not cigarette craving, and only showed up in the VAS data, not the TCQ data.

Finally, despite evidence from previous research [27], the aroma of the e-cig did not seem to affect the cigarette or e-cig craving.

Despite its strengths, this study also has a number of limitations. A first potential weakness is the fact that it is difficult to check whether the participants indeed did not smoke or vape for 12 h prior to the test session. A control system was built into the procedures, namely the measurement of CO, but this does not provide conclusive evidence. CO measures only give an indication of (the abstinence of) smoking regular cigarettes, not of vaping e-cigs, which does not influence the CO level [24]. The possible variance in the length of the actual periods of abstinence of both smoking and vaping can influence the baseline level of craving and possibly also the further course of post-vaping craving levels, which is one of our main dependent variables. This is especially problematic in the case where the actual abstinence periods may have been different between different experimental conditions.

A second potential weakness concerns our operationalization of the critical conditioned stimuli. We worked from the hypothesis that the characteristics of the e-cig resemble the conditioned stimuli of regular tobacco smoking. During the experiment, the participants noted, however, that the tobacco flavor of the e-cigs did substantially differ from the flavor of real tobacco, and especially of smoked tobacco. The question then becomes whether the flavors were sufficiently alike to generalize the conditioning effects from the tobacco flavor in combustible cigarettes to the tobacco flavor in e-cigs. This also implies that, in this study, the presentation of the tobacco flavor cues was only partially realized, which may at least in part explain the lack of effects of the aroma manipulation. It is possible that results would be different with another e-cig tobacco aroma, but with current vaping technology, it is very unlikely that the aroma of smoked tobacco would be replicated to perfection. With regard to the handling characteristics, a similar remark can be made. The e-cig is and was reported by the participants to be remarkably longer and especially heavier than a tobacco cigarette. Given this fact, the handling cues of the e-cigs also probably only partially mimicked the handling cues of a regular cigarette. Again, this can influence the degree of generalization of the conditioned handling aspects from smoking a tobacco cigarette to using an e-cig. Despite the fact that the cues were not perfectly simulated, the e-cig was a non-refillable “cig-a-like”, which offers a better approximation of the handling cues than the refillable second, third, or fourth generation e-cigs with larger and heavier batteries (i.e., box-mods). On the other hand, it has to be noted that some aspects of the handling, namely the “mouth action” and the stimuli resulting from the puffing (inhaling/exhaling), remained present even in the Unipod condition. This way, it is possible that some crucial aspects of the handling were still present in the condition that was intended to remove these stimuli. In this sense, our manipulation of the sensorimotor stimuli only allowed for a less than ideal assessment of the role of handling cues on craving reduction. Note that some studies did take away the handling completely by administering the nicotine intravenously [12,13].

A third potential weakness concerns the power of the present study. Investigating 16 conditions with 81 participants will have certain drawbacks, such as the fact that small effects cannot be found or that the found effects are potentially overestimated. Especially in the three-way interactions, sufficient power is an issue that should be taken into account.

A fourth potential weakness is the generalizability of the findings. Generalizing the findings to more prolonged/more frequent vaping in everyday situations should be done with great care since this study (a) took place in an artificial laboratory setting requiring repeated explicit craving reports and (b) implemented only a single five minute opportunity to vape in order to reduce craving with a one-hour follow-up. 

A final potential weakness concerns the duration of the period that participants could get acquainted with using an e-cig before the actual lab session. The literature is not consistent about the amount of time that is needed to provide the participants with sufficient experience with the e-cig [24,40]. Sufficient experience may be important, however, not only to attain optimal craving reduction but also to be able to demonstrate sufficient sensitivity to some subtle manipulations of the smoking/vaping-related CSs.

## 5. Conclusions

Our findings indicate that nicotine is an important variable in craving reduction. However, we also observed reliable craving reduction when the e-cig contained no nicotine, be it to a lesser degree than with nicotine. For one thing, this clearly demonstrates that the non-nicotine CSs that are present when using an e-cig do contribute to craving reduction. The findings also suggest a special role for sensorimotor handling cues: the superiority of nicotine-containing e-cigs over nicotine-free e-cigs was only observed when the handling cues were omitted. For the craving-reducing potential of other non-nicotine CSs, either no (aroma) or very weak and hard to interpret (visual cues) support was found in the present study. However, it should be noted that potential effects of (the presence or absence of) non-nicotine vaping cues largely depend on the specific operationalization of the (presence or absence of) critical conditioned stimuli, and to the extent to which vaping cues resemble those of (smoking) a tobacco cigarette. This similarity may have been compromised in the present study (e.g., flavor), and the operationalization of the presence/absence of some critical cues may well have been less than ideal (e.g., handling cues). Furthermore, due to the absence of a control condition assessing potential demand effects, the influence of the mere passage of time and/or of the repeated measurement procedure per se, definitive conclusions are not possible. Nevertheless, in the light of THR, these findings are relevant since they indicate which aspects of vaping may be important to focus on in order to imitate the smoking ritual as closely as possible.

## Figures and Tables

**Table 1:** Results of the mixed-effects models with respect to tobacco craving questionnaires (TCQ) and e-TCQ.

Outcome	Model	*χ*^2^ (df)	*p*	Condition	Pre vs. Post *	*t* (df)	*p*
TCQ	Moment	42.60 (1)	<0.01		−5.51	−7.48 (1)	<0.01
	Moment * Nicotine Level	9.48 (2)	<0.01				
				Nicotine	−7.75	−7.83 (1)	<0.01
				No-Nicotine	−3.34	−3.42 (1)	<0.01
	Moment * Nicotine Level * Handling	6.21 (4)	0.18				
	Moment * Nicotine Level * Aroma	3.95 (4)	0.41				
	Moment * Nicotine Level * Visual Cues	4.18 (4)	0.38				
e-TCQ	Moment	17.06 (1)	<0.01		−3.60	−4.38 (1)	<0.01
	Moment * Nicotine Level	7.17 (2)	<0.05				
				Nicotine	−5.57	−4.91 (1)	<0.01
				No-Nicotine	−1.68	−1.50 (1)	0.14
	Moment * Nicotine Level * Handling	1.40 (4)	0.85				
	Moment * Nicotine Level * Aroma	1.77 (4)	0.78				
	Moment * Nicotine Level * Visual Cues	7.57 (4)	0.11				

* Pre vs. Post means that Pre measurement was subtracted from Post measurements. Negative values indicate a higher Pre measurement in comparison to the post measurement.

**Table 2:** Results of the mixed-effects models with respect to visual analogue scale (VAS) and e-VAS.

Outcome	Index	Model	*χ*^2^ (df)	*p*	Condition	Pre vs. Post1 *	*t* (df)	*p*	Pre vs. Post1–5 *	*t* (df)	*p*
VAS	Moment		95.52 (5)	<0.01		−19.43	−8.31 (1)	<0.01	−14.88	−7.82 (1)	<0.01
	Moment * Nicotine Level		11.01 (6)	0.09							
					Nicotine	−26.76	−8.20 (1)	<0.01	−18.79	−7.95 (1)	<0.01
					No-Nicotine	−12.10	−3.66 (1)	<0.01	−10.97	−3.70 (1)	<0.01
	Moment * Nicotine Level * Aroma		5.99 (5)	0.31							
	Moment * Nicotine Level * Visual Cues		6.87 (5)	0.23							
	Moment * Nicotine Level * Handling		9.52 (5)	0.09							
	Moment * Nicotine Level by Manual		2.49 (6)	0.87							
	Moment * Nicotine Level by Unipod		20.52 (6)	<0.01							
e-VAS	Moment		37.79 (5)	<0.01		11.14	−4.77 (1)	<0.01	−7.07	−3.79 (1)	<0.01
	Moment * Nicotine Level		10.35 (6)	0.11							
					Nicotine	−15.42	−4.70 (1)	<0.01	−8.40	−3.55 (1)	<0.01
					No-Nicotine	−6.86	−2.10 (1)	<0.01	−5.75	−2.00 (1)	<0.05
	Moment * Nicotine Level * Aroma		3.73 (5)	0.59							
	Moment * Nicotine Level * Visual Cues		9.95 (5)	0.08							
	Moment * Nicotine Level by Blind		4.48 (6)	0.61							
	Moment * Nicotine Level by Unrestricted		15.75 (6)	<0.05							
	Moment * Nicotine Level * Handling		9.62 (5)	0.09							
	Moment * Nicotine Level at Manual		8.76 (6)	0.19							
	Moment * Nicotine Level at Unipod		11.92 (6)	0.06							

* Pre vs. Post means that we subtracted the Pre measurement from the Post measurement. Negative values indicate a higher Pre measurement in comparison to the post measurement.
